# Functional Genomics, Genetics, and Bioinformatics 2016

**DOI:** 10.1155/2016/2625831

**Published:** 2016-11-22

**Authors:** Youping Deng, Hongwei Wang, Ryuji Hamamoto, Shiwei Duan, Mehdi Pirooznia, Yongsheng Bai

**Affiliations:** ^1^Bioinformatics Core, Office of Biostatistics & Quantitative Health Sciences, Department of Tropical Medicine, Medical Microbiology, and Pharmacology, University of Hawaii John A. Burns School of Medicine, Honolulu, HI 96813, USA; ^2^Department of Medicine, University of Chicago, Chicago, IL 60637, USA; ^3^Division of Molecular Modification and Cancer Biology, National Cancer Center Research Institute, Tokyo, Japan; ^4^School of Medicine, Ningbo University, Ningbo, Zhejiang 315211, China; ^5^Bioinformatics and Computational Biology Core Facility, National Heart, Lung, and Blood Institute, Office of the Scientific Director, National Institutes of Health, Bethesda, MD 20814, USA; ^6^Department of Biology, Indiana State University, Terre Haute, IN 47809, USA

During the postgenomics era, more and more “omics” data are being produced due to rapid development of cutting-age technologies such as next generation sequencing. These omics data include genomics [1–3], transcriptomics [4–7], proteomics [8–10], metabolomics [11–13], and epigenomcis [14–16]. Bioinformatics plays a central role in analyzing and managing this huge amount of “omics” data, further understanding the function of biological molecules in different levels.

Several bioinformatics tools and methods have been developed in the special issue. C.-H. Tung et al. developed a new method called QuaBinggo, a prediction system for protein quaternary structure attributes using block composition. The method is 23% of Matthews Correlation Coefficient (MCC) higher than the existing prediction systems.* Exiguobacterium antarcticum* B7 is extremophile Gram-positive bacteria able to survive in cold environments. A key factor to understanding cold adaptation processes is related to the modification of fatty acids composing the cell membranes of psychrotrophic bacteria. R. Kawasaki et al. reconstructed the fatty acid biosynthesis pathway of* E. antarcticum* B7 based on both genomic and bibliomic data using bioinformatics methods, which is a great resource for the research of* Exiguobacterium antarcticum* B7. L. Hua and C. Quan built a novel method for protein-protein interaction (PPI) extraction using a shortest dependency path based CNN (sdpCNN) model. The proposed method only takes the sdp and word embedding as input and could avoid bias from feature selection by using CNN. The new approach outperformed traditional state-of-the-art kernel based methods. Clustered regularly interspaced short palindromic repeat (CRISPR) is a genetic element with active regulation roles for foreign invasive genes in the prokaryotic genomes and has been engineered to work with the CRISPR-associated sequence (Cas) gene Cas9 as one of the modern genome editing technologies. G. Mai et al. provided a valuable comprehensive curation resource to show the dynamic evolutionary patterns of prokaryotic CRISPRs based on computational evolutional analysis of 8 completely sequenced species in the genus* Thermoanaerobacter*.

Two papers are focused on transcriptomics data analyses. Q. Sun et al. tried to understand the gene function of thga1 in* Trichoderma harzianum* Th-33, important biocontrol filamentous fungi, which are widely used for their adaptability, broad antimicrobial spectrum, and various antagonistic mechanisms. Illumina RNA-seq technology (RNA-seq) was used to determine transcriptomic differences between the wild-type strain andthga1 mutant. A total of 888 genes were identified as differentially expressed genes (DEGs), including 427 upregulated and 461 downregulated genes. According to the functional annotation of these DEGs, they found the most abundant group was “secondary metabolite biosynthesis, transport, and catabolism.” Hepatitis E virus- (HEV-) mediated hepatitis has become a global public health problem. K. Xu et al. investigated the function of ORF3 from the swine form of HEV (SHEV); high-throughput RNA-Seq-based screening was conducted to identify the differentially expressed genes in ORF3-expressing HepG2 cells. The results indicated that, in the established ORF3-expressing HepG2 cells, the mRNA levels of CLDN6, YLPM1, APOC3, NLRP1, SCARA3, FGA, FGG, FGB, and FREM1 were upregulated, whereas the mRNA levels of SLC2A3, DKK1, BPIFB2, and PTGR1 were downregulated.

Several studies are concentrated on functional genomics data analyses. Using Web service SNP_TATA_Comparator presented in their previous paper, P. Ponomarenko et al. analyzed immediate surroundings of known SNP markers of diseases and identified 53 candidate SNP markers that can significantly change the affinity of TATA-binding protein for human gene promoters, with circadian consequences. These candidate SNP markers could be potentially useful for physicians (to select optimal treatment for each patient) and for the general population (to choose a lifestyle preventing possible circadian complications of diseases). M. Abu Saleh et al. conducted a comprehensive computational analysis on the functional and structural impacts of single nucleotide polymorphisms (SNPs) of the human ADIPOR1 at protein level. Their analyses suggested that the aforementioned variants, especially H341Y, could directly or indirectly destabilize the amino acid interactions and hydrogen bonding networks of ADIPOR1. Z.-X. Chen et al. used BLAST to call SNPs for non-model organisms based on 16 mixed functional gene's sequence data of polyploidy wheat. They demonstrated that mixed samples' NGS sequences and then analysis by BLAST were an effective, low-cost, and accurate way to mine SNPs for nonmodel species. Assembled reads and polynomial fitting threshold were recommended for more accurate SNPs targets.

One article deals with proteomics data analysis. S. Wan et al. indemnified important differential proteins between patients of slow transit constipation and normal controls using two-dimensional electrophoresis followed by laser desorption ionization tandem time-of-flight mass spectrometry (MALDI-TOF-MS). One paper is related to epigenomics data analysis. MiR-23a-27a-24-2 cluster has various functions and aberrant expression of the cluster is a common event in many cancers. Y. Wang et al. found a CG-rich region spanning two SP1 sites in the cluster promoter region. The SP1 sites in the cluster were demethylated and methylated in Hep2 cells and HEK293 cells, respectively. The demethylated SP1 sites in miR-23a-27a-24-2 cluster upregulate the cluster expression, leading to proliferation promotion and early apoptosis inhibition in laryngeal cancer cells.

Interaction of gene and environmental factors plays an important role in human diseases. Y. Wang et al. have found many important risk factors that affect chronic prostatitis/chronic pelvic pain syndrome (CP/CPPS), including biological, social, and psychological factors. They also discussed the potential interaction between genes and these risk factors.

In summary, this special issue presents a broad range of topics from functional genomics, transcriptomics, proteomics, epigenomics, and bioinformatics. It covers a variety of diseases such as cancer, hepatitis, chronic prostatitis, and infectious diseases. The study organisms include human, plant, and microorganisms. We hope that the readers will find interesting knowledge and methods in the issue.
